# Expression profiling of mouse embryonic fibroblasts with a deletion in the helicase domain of the Werner Syndrome gene homologue treated with hydrogen peroxide

**DOI:** 10.1186/1471-2164-11-127

**Published:** 2010-02-22

**Authors:** Adam Labbé, Ramachander VN Turaga, Éric R Paquet, Chantal Garand, Michel Lebel

**Affiliations:** 1 Centre de Recherche en Cancérologie de l'Université Laval, Hôpital Hôtel-Dieu de Québec, Québec, Canada

## Abstract

**Background:**

Werner Syndrome (WS) is a rare disorder characterized by the premature onset of a number of age-related diseases. The gene responsible for WS encodes a DNA helicase/exonuclease protein believed to affect different aspects of transcription, replication, and/or DNA repair. In addition to genomic instability, human WS cells exhibit oxidative stress. In this report, we have examined the impact of exogenous hydrogen peroxide on the expression profile of mouse embryonic fibroblasts lacking part of the helicase domain of the *WRN *homologue (here referred to as *Wrn*^Δ*hel*/Δ*hel*^).

**Results:**

*Wrn*^Δ*hel*/Δ*hel *^mutant mouse embryonic fibroblasts exhibit increased oxidative stress. This was reflected by increased intracellular reactive oxygen species (ROS), increased oxidative damage in genomic DNA, changes in ATP/ADP ratios, and a disruption of the inner mitochondrial transmembrane potential when compared to wild type mouse embryonic fibroblasts. Expression profile analyses of hydrogen peroxide-treated wild type cells have indicated significant decreases in the expression of genes involved in mitosis, glycolysis, fatty acid metabolism, nucleic acid metabolism, and cell cycle control, as well as protein modification and stability. Such decreases in these biological processes were not observed in hydrogen peroxide-treated *Wrn*^Δ*hel*/Δ*hel *^cells. Importantly, untreated *Wrn*^Δ*hel*/Δ*hel *^cells already exhibited down regulation of several biological processes decreased in wild type cells that had been treated with hydrogen peroxide.

**Conclusion:**

Expression profiling of *Wrn*^Δ*hel*/Δ*hel *^mutant cells revealed a very different response to exogenous addition of hydrogen peroxide in culture compared to wild type cells. This is due in part to the fact that *Wrn*^Δ*hel*/Δ*hel *^mutant cells already exhibited a modest chronic intracellular oxidative stress.

## Background

It is well established that increased levels of reactive oxygen species (ROS) are involved in a number of diseases including diabetes, complications from obesity, atherosclerosis, and cancer [1-3]. A major source of endogenous ROS comes from the mitochondria during the process of oxidative phosphorylation to produce energy in the form of ATP. In addition, ROS are produced by intracellular membrane oxidases following stimulation either with platelet-derived growth factors, TNF-α, or insulin [1-3]. Inflammation is also a major source of ROS at sites of tissue fibrosis [1-3]. It is thus important for the cell to rapidly neutralize ROS before they can damage cellular macromolecules including DNA. A major DNA lesion generated by excessive ROS is 8-oxo-2'-deoxyguanosine, which leads to a single or double strand break when left unrepaired [4]. Persistent breaks can in turn lead to genomic instability. It is widely believed that the accumulation of mutations is a main cause of several aging processes [5]. In addition, oxidative stress is known to shorten telomeres [6] a process likely leading to replicative senescence and aging as well [7]. Thus, an abnormal response to constant increased levels of endogenous intracellular ROS would likely affect aging [8,9].

Some specific inherited monogenic diseases appear to modulate multiple aspects of aging. They are referred as segmental progeroid syndromes. A common feature of all progeroid syndromes is genomic instability. One such syndrome is Werner syndrome (WS) also known as "Progeria of the Adult" [10]. WS is an autosomal recessive disorder characterized by genomic instability and the premature onset of a number of age related diseases [11,12]. The gene responsible for WS (*WRN*) was identified by positional cloning and encodes a protein containing a RecQ-type helicase consensus domain [13]. It was subsequently found that, in addition to a 3'-5' helicase activity, the WRN protein also possesses a 3'-5' exonuclease activity [14,15]. It has been recently proposed that WRN protein may be required for the repair of oxidative DNA damage [16] including oxidative DNA damage at telomeres [17].

Remarkably, increased oxidative stress was described for WS patients [18]. Furthermore, human WS fibroblasts exhibit increased intracellular oxidized protein content [19]. Increased oxidative stress was also observed in embryonic cells derived from mice lacking part of the helicase domain [20]. In this study, we sought to determine the impact of additional oxidative stress in mouse embryonic fibroblasts lacking part of the helicase domain of the *WRN *gene ortholog. Expression profiling analyses of *Wrn*^Δ*hel*/Δ*hel *^mutant cells revealed a very different response to addition of hydrogen peroxide in culture compared to wild type cells as they already exhibit a modest but significant chronic increase in intracellular ROS levels.

## Results

### Oxidative stress in *Wrn*^Δ*hel*/Δ*hel *^mouse embryonic fibroblasts

**Figure 1:**
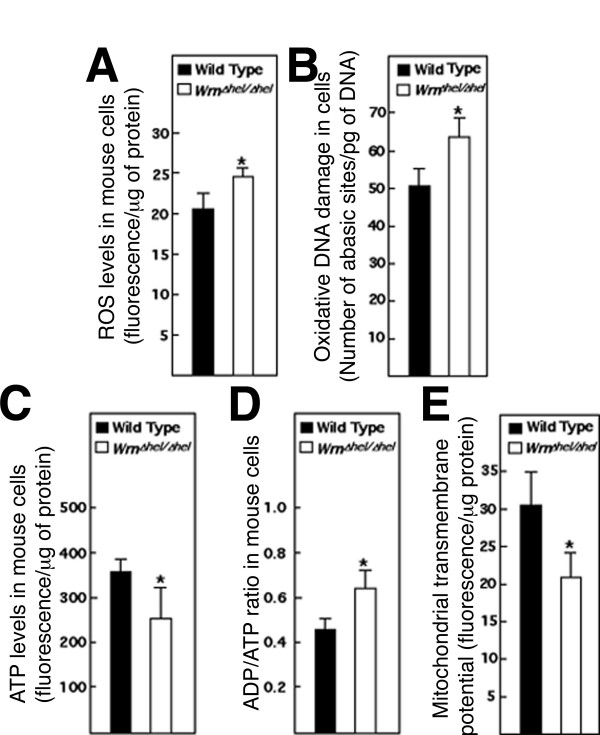
**Increased oxidative stress in mutant mouse embryonic fibrobasts**. (A) ROS levels in wild type and *Wrn*^Δ*hel*/Δ*hel *^cells determined by measuring the intensity of fluorescence by 2',7'-dichlrofluorescein per μg of protein in cells. Asterisks denote statistical significance compared to wild type cells (*t-test: *P *< 0.05). (B) Oxidative DNA lesions created by ROS in wild type and *Wrn*^Δ*hel*/Δ*hel *^cell cultures. The number of abasic sites per pg of genomic DNA was detected as described in Materials and Methods. The asterisk denotes statistical significance compared to wild type cells (*t-test: *P *< 0.05). (C) ATP levels in wild type and *Wrn*^Δ*hel*/Δ*hel *^cells. Asterisks denote statistical significance compared to wild type cells (*t-test: *P *< 0.05). (D) ADP/ATP ratios in wild type and *Wrn*^Δ*hel*/Δ*hel *^cells. Asterisks denote statistical significance compared to wild type cells (*t-test: *P *< 0.05). (E) Inner mitochondrial transmembrane potential in wild type and *Wrn*^Δ*hel*/Δ*hel *^cells. Mitochondrial membrane potential was measured with the potentiometric dye TMRE. Asterisks denote statistical significance compared to wild type cells (*t-test: *P *< 0.03). All experiments in this figure were performed in quadruplicate.

Previous data have indicated that *Wrn*^Δ*hel*/Δ*hel *^mouse embryonic fibroblasts acquire a slower growth rate than wild type fibroblasts with the number of passages in culture [21]. In addition, *Wrn*^Δ*hel*/Δ*hel *^mutant cells exhibit increased chromosomal rearrangements with the number of passages in culture [22]. As oxidative DNA damage can lead to chromosomal rearrangements, levels of intracellular ROS and the extent of oxidative DNA damage were measured in wild type and *Wrn*^Δ*hel*/Δ*hel *^embryonic fibroblasts. Intracellular ROS levels were examined directly in mouse embryonic fibroblasts with the dye 2'-7' dichlorofluorescein diacetate. This dye is highly fluorescent upon oxidation. Cells in the presence of the dye were lysed and the extent of fluorescence released from cells was measured with a fluorescence spectrophotometer as described previously [23]. As shown in Figure 1A, ROS levels were 10% higher in *Wrn*^Δ*hel*/Δ*hel *^cells (*t*-test; *P *< 0.05) compared to wild type cells. Concomitantly, there were on average 51 abasic sites per pg of genomic DNA in wild type cells and 64 abasic sites per pg of genomic DNA in *Wrn*^Δ*hel*/Δ*hel *^cells, a 20% increase in the mutant cells (*t*-test; *P *< 0.05) (Figure 1B). These results indicate that *Wrn*^Δ*hel*/Δ*hel *^cells exhibit a modest but significant increase in oxidative stress.

Since a cellular redox change may decrease energy production in the form of ATP from mitochondria, we next examined intracellular ATP levels and mitochondrial membrane potential. As shown in Figure 1C, the ATP level in *Wrn*^Δ*hel*/Δ*hel *^mutant cells was 30% lower than wild type cells. Consequently, the ADP/ATP ratio in *Wrn*^Δ*hel*/Δ*hel *^cells was approximately 30% higher than wild type cells (Figure 1D). A lower ATP production might be a consequence of disruption of the inner mitochondrial transmembrane potential in mutant cells. The mitochondrial membrane potential was thus examined in our mouse embryonic fibroblasts with the fluorescent dye tetramethylrhodamine ethyl ester (TMRE) As indicated in Figure 1E, fluorescence intensity was 30% lower in *Wrn*^Δ*hel*/Δ*hel *^cells compared to wild type cells (*t*-test; *P *< 0.03). Taken together, these results indicate a disturbance of mitochondrial activities in *Wrn*^Δ*hel*/Δ*hel *^mutant cells.

**Figure 2:**
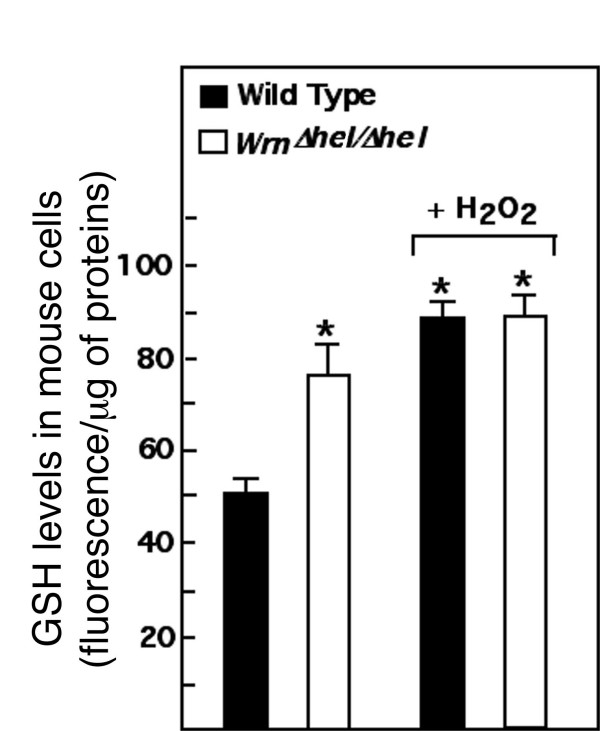
**Reduced GSH levels in H_2_O_2_-treated and untreated wild type and *Wrn*^Δ*hel*/Δ*hel *^mouse embryonic fibroblasts determined by the intensity of fluorescence per μg of protein**. Fluorescence of reduced GSH was detected with the ApoGSH glutathione detection kit (Bio Vision Mountain View, CA). Oxidative stress was induced by treating cells with 0.5 mM H_2_O_2 _one hour and fresh medium was then added for an additional 24 hours to allow the cells to recover before measurements. (*t-test: *P *< 0.001 compared to untreated wild type mouse embryonic fibroblasts).

Glutathione (GSH) is the principal intracellular low-molecular-weight thiol and it plays a critical role in the defense against oxidative stress in mammalian cells. We wanted to know whether the abundance of this molecule is affected in mutant cells. Levels of GSH were thus quantified in our mouse wild type and *Wrn*^Δ*hel*/Δ*hel *^mutant embryonic fibroblasts. Remarkably, levels of GSH were 151% higher in *Wrn*^Δ*hel*/Δ*hel *^mutant cells compared to wild type cells (*t*-test; *P *< 0.001) (Figure 2). These results suggest that *Wrn*^Δ*hel*/Δ*hel *^mutant cells display an adaptive response to oxidative stress. This response was further examined by treating cells with exogenous H_2_O_2_. Cells were treated one hour with 0.5 mM H_2_O_2 _and GSH levels were measured 24 hours later. As indicated in Figure 2, H_2_O_2 _induced a 178% increase in GSH levels in wild type cells (*t*-test; *P *< 0.001). There was no significant increase in GSH levels in *Wrn*^Δ*hel*/Δ*hel *^mutant cells. As indicated above GSH levels were already high in untreated *Wrn*^Δ*hel*/Δ*hel *^cells. These results indicate that *Wrn*^Δ*hel*/Δ*hel *^mutant cells continuously exhibited an increased anti-oxidant response.

### Expression profiling of mouse embryonic fibroblasts treated with H_2_O_2_

Little is known about the global response to H_2_O_2 _in wild type and *Wrn*^Δ*hel*/Δ*hel *^mouse embryonic fibroblasts. To compile a list of H_2_O_2_-responsive genes, we compared wild type cells before and after oxidative cellular stress. Cells were treated with H_2_O_2 _for one hour, fed with fresh media, and cytoplasmic RNA was harvested 24 hours later. Hybridization was performed on Whole Mouse Genome Agilent 60-mer Oligo Microarray chips (containing approximately 44,000 probes) by mixing wild type Cy-3-labeled cRNA (baseline expression levels) with H_2_O_2_-treated wild type Cy-5-labeled cRNA. Hybridization experiments were performed twice with different wild type biological replicates. The same strategy was adopted for *Wrn*^Δ*hel*/Δ*hel *^mutant cells. Hybridization was performed by mixing untreated *Wrn*^Δ*hel*/Δ*hel *^mutant Cy-3-labeled cRNA with H_2_O_2_-treated *Wrn*^Δ*hel*/Δ*hel *^Cy-5-labeled cRNA.

**Figure 3:**
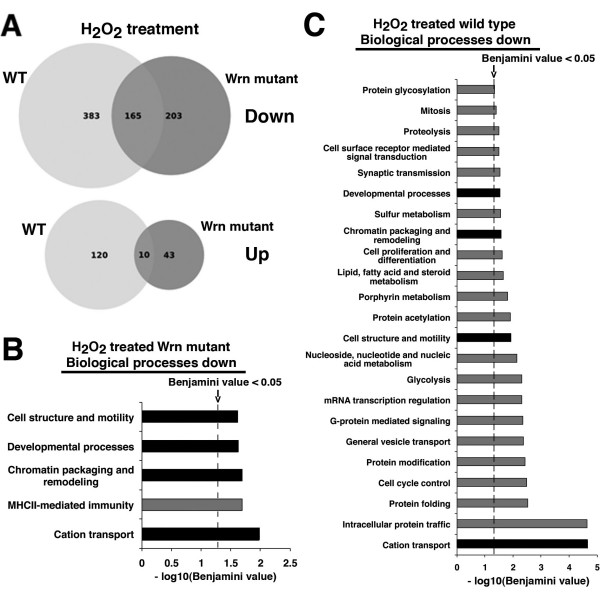
**Distinct biological processes significantly altered by H_2_O_2 _in *Wrn*^Δ*hel*/Δ*hel *^and wild type mouse embryonic fibroblasts**. (A) Venn diagrams showing the number of genes overlapping between *Wrn*^Δ*hel*/Δ*hel *^and wild type mouse embryonic fibroblasts treated with H_2_O_2_. (B) Histogram representing all the biological processes significantly down regulated by H_2_O_2 _in *Wrn*^Δ*hel*/Δ*hel *^mouse embryonic fibroblasts. (C) Histogram representing all the biological processes significantly down regulated by H_2_O_2 _in wild type mouse embryonic fibroblasts. Biological processes with a Benjamini value less than 0.05 are presented in each histogram (as the -log10 of the Benjamini value). The black bars in each histogram represent the cellular processes commonly affected in both *Wrn*^Δ*hel*/Δ*hel *^and wild type mouse embryonic fibroblasts.

Lists of genes differentially expressed were generated by requiring that the absolute value of the fold change be higher than two, that the log2 of the expression level (or signal) for each gene be higher than six, and the adjusted *P*-value, using the Benjamini-Hochberg method, be lower than 0.005. Six hundred and seventy eight genes exhibited a two-fold alteration in mRNA expression level in H_2_O_2_-treated wild type mouse embryonic fibroblasts compared to untreated wild type cells (548 down-regulated and 130 up-regulated, respectively) (additional file 1). Four hundred and twenty one genes exhibited a two-fold alteration in expression in H_2_O_2_-treated *Wrn*^Δ*hel*/Δ*hel *^mouse embryonic fibroblasts compared to untreated *Wrn*^Δ*hel*/Δ*hel *^cells (368 down-regulated and 53 up-regulated, respectively) (additional file 2). One hundred and seventy five genes were altered in both H_2_O_2_-treated wild type and *Wrn*^Δ*hel*/Δ*hel *^mouse embryonic fibroblasts (Figure 3A and additional file 3).

Real time RT-PCR was performed on seven randomly picked genes from the untreated versus H_2_O_2_-treated wild type mouse embryonic fibroblasts data (additional file 1) to confirm our microarray analyses (Table 1). A list of the primers for the genes analyzed is shown in the additional file 4. Overall, the fold changes observed by real time RT-PCR were in good agreement with the predicted fold changes from the microarray data highlighted by a significant Spearman's correlation (rho = 0.9286; *P *= 0.0067).

**Table 1:** Correlation of the real time RT-PCR-derived values with the microarray data from wild type mouse embryonic fibroblasts treated with peroxide.

Gene	Fold change in microarray	Fold change from RT-PCR
Ero1L (endoplasmic reticulum oxidoreductase 1-like)	+2.84	+2.76
Fn1 (fibronectin-1)	-2.19	-1.45
Mt1 (metallomethionin-1)	+2.25	+1.71
Mt2 (metallomethionin-2)		
Nqo1 (NAD(P)H dehydroxygenase, quinone 1)	+2.68	+1.65
Txnrd1 (thioredoxin reductase 1)	+2.06	+1.63
Uchl1 (ubiquitin carboxy-terminal hydrolase L1)	+3.72	+7.97

Spearman's correlation between microarray and RT-PCR data: rho = 0.9286; *p*-value = 0.0067

The PANTHER classification system was used to classify differentially expressed genes by their functions in specific biological processes. Categorization of the genes in our lists revealed 23 and 5 significantly down regulated biological processes (with a Benjamini value of less than 0.05) for wild type and *Wrn*^Δ*hel*/Δ*hel *^mouse embryonic fibroblasts treated with H_2_O_2_, respectively (Figure 3). Four processes were commonly down regulated in wild type and *Wrn*^Δ*hel*/Δ*hel *^mouse embryonic fibroblasts treated with H_2_O_2 _and they include genes involved in cation transport, chromatin remodeling, cell motility, and developmental processes (or cellular differentiation) (Figure 3B and 3C). The only additional biological process that was down regulated in *Wrn*^Δ*hel*/Δ*hel *^mouse embryonic fibroblasts corresponded to the cellular defense response requiring antigen presentation during major histo-compatibility complex II molecule (MHCII)-mediated immunity. Wild type mouse embryonic fibroblasts treated with H_2_O_2 _also exhibited decreases in biological pathways affecting protein and vesicle transportation, cell surface mediated signal transduction, cell cycle and proliferation, nucleic acid metabolism, several types of protein modification and folding, proteolysis, glycolysis, and mitosis (Figure 3C). Finally, based on PANTHER analysis no biological pathway was significantly up regulated in either H_2_O_2_-treated wild type or H_2_O_2_-treated *Wrn*^Δ*hel*/Δ*hel *^mouse embryonic fibroblasts.

### Several biological processes are already decreased in untreated *Wrn*^Δ*hel*/Δ*hel *^mouse embryonic fibroblasts

**Figure 4:**
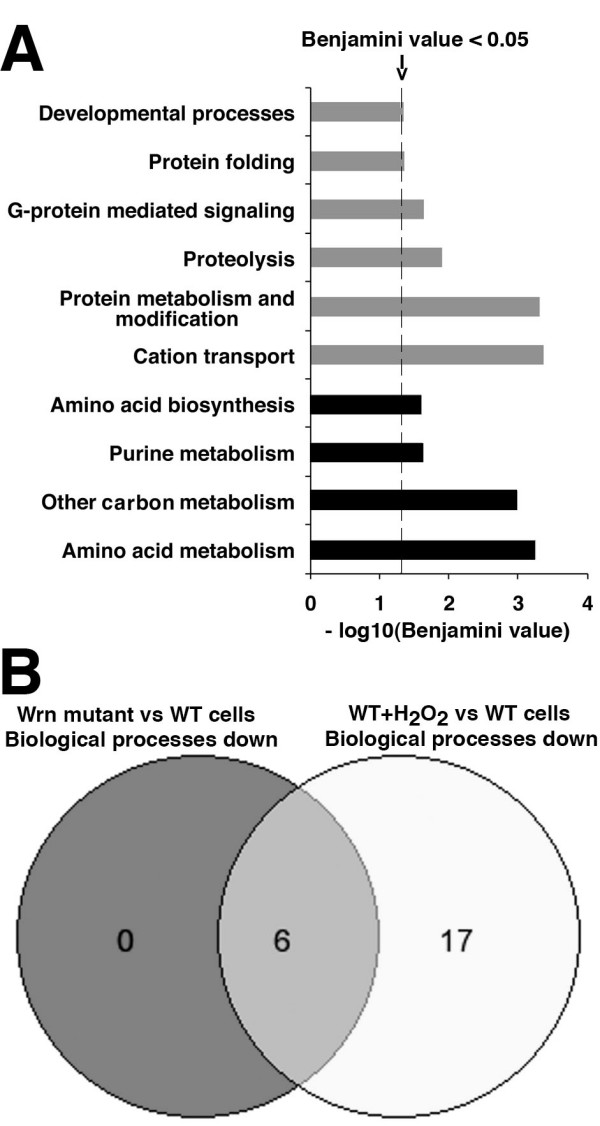
**Histogram indicating the major biological processes in untreated *Wrn*^Δ*hel*/Δ*hel *^mouse embryonic fibroblasts significantly changed compared to untreated wild type cells**. (A) The biological processes with a Benjamini value less than 0.05 are presented for untreated *Wrn*^Δ*hel*/Δ*hel *^mouse embryonic fibroblasts versus untreated wild type cells (baseline level) as the -log10 of the Benjamini value. The black and gray bars represent the up and down regulated cellular processes, respectively. (B) Venn diagrams showing the number of down regulated biological processes overlapping between untreated *Wrn*^Δ*hel*/Δ*hel *^and H_2_O_2_-treated wild type mouse embryonic fibroblasts.

We also performed PANTHER analysis on the list of genes differentially expressed between untreated wild type and untreated *Wrn*^Δ*hel*/Δ*hel *^mouse embryonic fibroblasts. Differentially expressed genes between *Wrn*^Δ*hel*/Δ*hel *^and wild type mouse embryonic fibroblasts were selected by requiring that the log2 of the expression level for each gene be higher than six and the adjusted *P*-value, using the Benjamini-Hochberg method, be lower than 0.005 (additional file 5). Figure 4A indicates that biological processes such as protein folding, protein metabolism and modification, proteolysis, G-protein-mediated signaling, cation transport, and developmental processes were significantly down regulated in *Wrn*^Δ*hel*/Δ*hel *^mouse embryonic fibroblasts compared to wild type cells (with a Benjamini value < 0.05). Purine metabolisms, as well as amino acid metabolism and biosynthesis processes, were up regulated in *Wrn*^Δ*hel*/Δ*hel *^mouse embryonic fibroblasts compared to wild type cells. These results indicate that all the biological processes down regulated in untreated *Wrn*^Δ*hel*/Δ*hel *^mouse embryonic fibroblasts (compared to untreated wild type cells) were also down regulated in H_2_O_2_-treated wild type mouse embryonic fibroblasts (Figure 4B).

## Discussion

We have previously observed increased oxidative stress in mouse embryonic fibroblasts established from *Wrn*^Δ*hel*/Δ*hel *^mice compared to wild type cells [20]. In this study, we further explored this phenotype by examining the expression profile of such cells. *Wrn*^Δ*hel*/Δ*hel *^mutant cell lines exhibited a 10% increase in ROS levels compared to wild type cells (baseline level). Importantly, the high levels of GSH in *Wrn*^Δ*hel*/Δ*hel *^mutant cells confirmed indirectly the increased oxidative stress in such cells. In fact, GSH levels in *Wrn*^Δ*hel*/Δ*hel *^mutant cells were almost as high as wild type cells that had been treated with exogenous H_2_O_2 _(Figure 2). Further analysis of cells with the potentiometric dye TMRE has indicated a lower mitochondrial transmembrane potential in *Wrn*^Δ*hel*/Δ*hel *^mutant cells. At the same time, we detected a lower amount of ATP in the mutant cells compared to wild type cells. Production of lower quantities of ATP by mitochondria may in part be responsible for the slow growth phenotype associated with *Wrn*^Δ*hel*/Δ*hel *^cells [20]. The increase in ROS concomitant with the high levels of GSH and the lower energy production (ATP) by mitochondria in *Wrn*^Δ*hel*/Δ*hel *^mouse embryonic fibroblasts are consistent with the phenotypes observed in the liver tissue of *Wrn*^Δ*hel*/Δ*hel *^mice [24]. *Wrn*^Δ*hel*/Δ*hel *^mice exhibit increased GSH levels in liver tissue with increased ROS, DNA damage and decrease ATP levels with age. These results suggest that the observed anomalies in the *Wrn*^Δ*hel*/Δ*hel *^mice are observable at the cellular level with mouse embryonic fibroblasts in culture. Such results suggest that cells in a given tissue already exhibit a modest but chronic intracellular pro-oxidative status. In accord with this, a pro-oxidative status has been described for WS patients [18].

Our prediction was that an exogenous addition of ROS to *Wrn*^Δ*hel*/Δ*hel *^mutant cells would exacerbate the intracellular metabolic anomalies and damage as the anti-oxidative system of such cells is already saturated (at least for the GSH system) while coping with a chronic higher intracellular level of ROS. The results obtained with mouse embryonic fibroblasts treated with an exogenous source of H_2_O_2 _are highly relevant since additional ROS at the tissue level may follow each meal due to increased sugar and lipid levels in the blood stream after ingestion. Increased sugar and lipids will potentially impact on the cellular redox status of cells in several tissues [25,26].

The impact of H_2_O_2 _on the expression profile of Wrn mutant cells has not been previously analyzed. Microarray analyses indicated that *Wrn*^Δ*hel*/Δ*hel *^mouse embryonic fibroblasts respond very differently from wild type cells to exogenous H_2_O_2_. These results are consistent with the observation that human WS cells respond differently to H_2_O_2 _when compared to cells with a functional WRN gene product [27,28]. Again, this difference maybe due to the fact that cells with a mutation in the *Wrn *gene are already exhibiting chronic increased oxidative stress in culture [20,29]. Significant decreases in the expression of genes involved in mitosis, glycolysis and fatty acid metabolism (energy production), nucleic acid metabolism, cell cycle control, as well as protein modification and stability were observed in H_2_O_2_-treated wild type embryonic fibroblasts. Such dramatic decreases in these biological processes were not observed in H_2_O_2_treated *Wrn*^Δ*hel*/Δ*hel *^cells. Interestingly, untreated *Wrn*^Δ*hel*/Δ*hel *^cells already exhibited changes in several of these biological processes before the addition of H_2_O_2 _suggesting again a chronic oxidative stress in these mutant cells. A likely deleterious consequence of responding differently to additional ROS is a greater increase in the presence of persistent DNA breaks and the rapid accumulation of chromosome rearrangements compared to wild type mouse embryonic fibroblasts [22].

The International Registry of Werner Syndrome http://www.wernersyndrome.org has provided molecular diagnosis of WS for over a decade. Fifty distinct mutations inactivating the WRN protein have been described in WS patients to date [30]. These mutations include missense and nonsense substitutions, frame shifts and premature translation termination mutations, deletions and insertions. All these mutations are believed to disrupt the normal function of WRN protein or to cause a truncation of the protein that cannot localize to the nucleus, the normal site of WRN protein action. Some mutations also cause abnormally rapid degradation of the WRN protein. Our mouse model has an in-frame deletion in the helicase domain of the Wrn protein, which is as stable as the normal protein [31]. However, the mutant protein does not stably interact with the DNA replication complex [32] suggesting that this mutation in mouse severely affects the overall function of the mutant Wrn protein. Although thorough biochemical analyses of this mutant protein are required, the *Wrn *helicase mutant mice provide a compelling model as they exhibit most metabolic abnormalities observed in human WS patients [23,24].

## Conclusion

To conclude, untreated *Wrn*^Δ*hel*/Δ*hel *^mutant cells exhibit increased ROS generation due to alterations in mitochondrial activities. The exact reason for such mitochondrial alterations is unknown but mitochondrial dysfunction is well known to lead to physiological decline with age [9]. We recently observed that a transient knock down of WRN protein in human fibroblasts is sufficient to change the expression of genes involved in lipidogenesis [33]. Such changes are known to affect the redox status in cells, which in turn affects mitochondrial function [34]. Interestingly, we have also observed that *Wrn*^Δ*hel*/Δ*hel *^mutant mice exhibit increased rate of point mutations in the mitochondrial DNA of liver and heart tissues [24], which can potentially lead to further mitochondrial dysfunction with age. Finally, a transient knock down of WRN in human fibroblasts will increase the activity of several protein kinase C enzymes including PKCδ [29]. PKCδ is known to modulate ROS production from mitochondria [35]. Hence, there is the interesting possibility that depletion of WRN activates PKCδ, which in turn modulates ROS production from mitochondria. Indeed, a decreased production of ROS in WRN-depleted fibroblasts can be observed by knocking down PKCδ protein levels in such cells. Future appropriate experiments will determine which pathways, transcription regulation of specific metabolic genes or activation of specific kinases, are responsible for the redox imbalance seen in human WRN mutant or mouse *Wrn*^Δ*hel*/Δ*hel *^cells.

## Methods

### Primary mouse embryonic fibroblasts and RNA

Mice lacking part of the helicase domain of the *Wrn *gene were generated by homologous recombination in mouse embryonic stem cells [31]. In the process, 121 amino acid residues of the Wrn protein were deleted (amino acids 710 to 831). The genetic background of these mice was both 129/Sv and Black Swiss (129/Sv/Black Swiss genetic background). Care of mice was in accordance with the guidelines of the Committee for the protection of animals at the Université Laval. Generation and maintenance of the embryonic cells has been described previously [21]. Briefly, healthy 15.5-day old embryos were minced in 6-well plates and maintained in low glucose DMEM supplemented with 10% heat-inactivated calf serum at 37°C in an atmosphere of 5% CO_2_. Adherent cells established from embryonic tissues were passaged as soon as they reached confluence. Cytoplasmic RNA was extracted according to standard protocols [36]. After DNAse treatment and phenol:chloroform extraction, precipitated cytoplasmic RNA was dissolved in RNase-free water and purity was verified with an Agilent 2100 bioanalyzer (Agilent, Palo Alto, CA).

### Microarray analysis

Duplicated biological RNA samples (wild type versus H_2_O_2_-treated wild type; *Wrn*^Δ*hel*/Δ*hel *^versus H_2_O_2_-treated *Wrn*^Δ*hel*/Δ*hel *^cells; wild type versus *Wrn*^Δ*hel*/Δ*hel*^) were labeled with Cyanin-3 or -5 labeled CTP (PerkinElmer, Boston, MA). Labeled cRNAs were purified using the RNeasy Mini kit (Qiagen, Mississauga, ON) and were hybridized onto Whole Mouse Genome Agilent 60-mer Oligo Microarrays (44,000 probes/microarray) using the in situ Hybridization Plus kit (Agilent, Palo Alto, CA) following the manufacturer's instructions. Arrays were scanned using a dual-laser DNA microarray scanner. Data were then extracted from images by the Feature Extraction software 6.1 (Agilent, Palo Alto, CA). Lists of differentially expressed genes were generated using limma in BioConductor http://www.bioconductor.org. The data were background subtracted and normalized using the loess method. Correction for multiple hypothesis testing was performed using the Benjamini-Hochberg method. We have deposited all the raw data in the NCBI public database Gene Expression Omnibus http://www.ncbi.nlm.nih.gov/geo/ [accession series number GSE19007].

### Bioinformatic analyses

The PANTHER (Protein ANalysis THrough Evolutionary Relationships) classification system is a unique resource that classifies genes by their functions, using published scientific experimental evidence and evolutionary relationships to predict function even in the absence of direct experimental evidence. Proteins are classified by expert biologists into families and subfamilies of shared function, which are then categorized by molecular function and biological process ontology terms. This program is implemented in the DAVID (Database for Annotation, Visualization, and Integrated Discovery) web site [37]. Enrichments for specific biological functions using PANTHER were considered significant with a Benjamini value smaller than 0.05. The Benjamini value corresponds to an adjusted *p*-value using the Benjamini-Hochberg method to correct for multiple hypotheses tested during gene enrichment analyses.

### Measurements of reactive oxygen species

Mouse embryonic fibroblasts were incubated with 10 μg/μl of the dye 2'-7' dichlorofluorescein diacetate (Sigma-Aldrich Canada Ltd., Oakville, ON) for one hour at 37°C. This dye is highly fluorescent upon oxidation. After this incubation time, cells were harvested and lysed in RIPA buffer [50 mM Tris-HCl (pH 7.5), 150 mM NaCl, 1% NP-40, 0.1% SDS, 0.5% sodium deoxycholate] for 10 minutes. Cell debris were spun down and 100 μl of lysate was transferred to 96-well plates for fluorescence measurements with a Fluoroskan Ascent fluorescence spectrophotometer (Thermo Electron Inc., Milford, MA). The excitation and emission wavelengths used were 485 nm and 527 nm, respectively. The final result was expressed as units of fluorescence per μg of proteins. Protein concentrations were measured using the Bradford assay.

### Measurements of oxidative DNA damage

DNA was extracted from mouse embryonic fibroblasts with the DNeasy Tissue Kit (Qiagen Inc., Mississauga, ON) according to the manufacturer's protocol. Abasic sites in genomic DNA were quantified with the Oxidative DNA Damage Kit from Kamiya Biomedical Company (Seattle, WA). Briefly, this kit contains an aldehyde reactive reagent (N'-aminooxymethylcarbonylhydrazino-D-biotin), which reacts with the open ring form of apurinic sites in DNA. Oxidative attack by hydroxyl radicals on the deoxyribose moiety will lead to the release of free bases from DNA, generating abasic or apurinic sites. The aldehyde reagent will thus tag apurinic sites with biotin. The number of biotin-tagged apurinic sites was quantified using conjugated avidin-horse radish peroxidase followed by a colorimetric detection of peroxidase.

### Measurements of reduced glutathione

Intracellular levels of glutathione (GSH) were quantified with the ApoGSH Glutathione Detection Kit from Bio Vision (Mountain View, CA). The assay utilizes monochlorobimane (MCB), a dye that appears to form adducts exclusively with GSH. The GSH-bound MCB dye fluoresces blue. Fluorescence was measured with a Fluoroskan Ascent fluorescence spectrophotometer (Thermo Electron Inc., Milford, MA). The excitation and emission wavelengths used were 355 nm and 460 nm, respectively.

### Measurements of ATP and ADP

Intracellular levels of ATP and ADP were quantified with the ApoSensor ADP/ATP ratio assay kit from Bio Vision (Mountain View, CA). The assay utilizes the enzyme luciferase to catalyze the formation of light from ATP and luciferin. ADP levels are measured by conversion to ATP that is subsequently detected using the same reaction. Luminescence was measured with a Luminoskan Ascent luminometer (Thermo Electron Inc., Milford, MA).

### Measurements of inner mitochondrial transmembrane potential

The inner mitochondrial transmembrane potential of mouse embryonic fibroblasts was determined using tetramethylrhodamine ethyl ester (TMRE). Cells were incubated in a culture medium containing 100 μM TMRE for 15 min in the dark at 37°C. Fresh medium was then added after washing cells twice with PBS to remove extracellular TMRE. Cells were harvested and fluorescence intensity of TMRE was detected with a Fluoroskan Ascent fluorescence spectrophotometer (Thermo Electron Inc., Milford, MA). The excitation and emission wavelengths used were 544 nm and 590 nm, respectively.

### Statistical analysis

Data are presented as means ± SEM. The unpaired Student's *t*-tests were all performed using two-tailed hypothesis and equal variance. Differences between classes were considered significant at *P*-value lower than 0.05 in all statistical analyses. All statistical analyses were performed with R version 2.6.0 http://www.r-project.org.

## Competing interests

The authors declare that they have no competing interests.

## Authors' contributions

All authors contributed to the overall experimental design. AL performed the RNA extraction and the real time RT-PCR experiments. RVNT established the mouse embryonic fibroblasts and performed the TMRE and ROS measurements. EP performed all statistical analyses. CG performed the DNA damage and GSH measurements. ML drafted the manuscript. All authors read, contributed to, and approved the final manuscript.

## Supplementary Material

Additional file 1 **Differential expression profile between untreated and H_2_O_2_-treated wild type mouse embryonic fibroblasts**. This table is listing the genes altered more than 2 times with a Benjamini-Hochberg adjusted p-value lower than 0.1 while comparing the H_2_O_2_ treated versus non-treated wild type mouse embryonic fibroblasts. Complete results of microarray analyses are found in additional files 1, 2, 4, and 5. Click here for file

Additional file 2 **Differential expression profile between untreated and H_2_O_2_-treated Wrn helicase mutant mouse embryonic fibroblasts**. This table is listing the genes altered more than 2 times with a Benjamini-Hochberg adjusted p-value lower than 0.1 while comparing the H_2_O_2_ treated versus non-treated Wrn helicase mutant mouse embryonic fibroblasts. Complete results of microarray analyses are found in additional files 1, 2, 4, and 5. Click here for file

Additional file 3 **Genes commonly altered in both H_2_O_2_-treated wild type and Wrn helicase mutant mouse embryonic fibroblasts**. This table is listing the common significant genes altered in the same direction in Wrn mutant and wild type mouse embryonic fibroblasts. The primers used for the real time RT-PCR analyses are shown in the additional file 3. Click here for file

Additional file 4 **Primers used for real time RT-PCR**. This table contains the sequence of the oligonucleotides that were used for the real time RT-PCR with the reaction conditions. Complete results of microarray analyses are found in additional files 1, 2, 4, and 5. Click here for file

Additional file 5 **Differential expression profile between wild type and Wrn helicase mutant mouse embryonic fibroblasts**. This table is listing the genes altered more than 2 times with a Benjamini-Hochberg adjusted p-value lower than 0.1 while comparing the Wrn mutant versus the wild type mouse embryonic fibroblasts. Complete results of microarray analyses are found in additional files 1, 2, 4, and 5. Click here for file
